# Dietary branched-chain amino acids enhance skeletal and intestinal development through targeted modulation of bone metabolism, tight junction proteins and amino acid transporter gene expression in broiler chickens

**DOI:** 10.1016/j.psj.2026.107448

**Published:** 2026-07-16

**Authors:** Shahriyar Khalilzadeh, Abolfazl Zarei, Nima Eila

**Affiliations:** Department of Animal Sciences, Ka. C., Islamic Azad University, Karaj, Iran

**Keywords:** BCAAs, Bone, Morphology, Gene expression, Nutrient transporters

## Abstract

Nutritional strategies to reduce dietary protein without impairing growth are essential in modern poultry production, as inadequate supply disrupts bone integrity, intestinal function, and nutrient utilization. The present study aimed to investigate the effects of using different levels of branched-chain amino acids (**BCAA**) in low crude protein (**CP**) diets on performance, skeletal development, intestinal morphology, and expression of genes related to bone metabolism, tight junction proteins, and amino acid transporter in broiler chickens. A total of 480 one-day-old male and female broiler chicks of the Ross 308 strain were used in a completely randomized design with 4 treatments and 6 replications. The experimental diets included a standard protein diet (**NCP**), a diet with 10% less protein than the standard (**LCP**), an LCP diet with 10% more BCAA than the requirement (LCP+10BCAA), and an LCP diet with 20% more BCAA than the requirement (LCP+20BCAA). Supplementation with BCAA at 10 and 20% levels improved body weight gain compared to the LCP treatment (P=0.03). The inclusion of 20% more BCAA than the requirements of the Ross strain in LCP diets increased tibial weight, strength, ash, and calcium content (P<0.05). The muscularis layer thickness was markedly greater in both BCAA-supplemented groups compared to NCP and LCP (P=0.001). However, villus height and villus surface area in broilers of the LCP + 20BCAA group were significantly higher than those of birds in the NCP, LCP, and LCP + 10BCAA groups (P<0.05). The mRNA expression of *RUNX-2, BGLAP*, and *BMP-2* genes was significantly increased in both LCP + 10BCAA and LCP + 20BCAA groups compared with LCP and NCP (P<0.05). *CLDN-1, ZO-1* and *MUC-2* expression was significantly upregulated in BCAA-supplemented groups compared with NCP and LCP (P<0.05). *SLC3A1* expression was significantly decreased in the LCP group compared to other groups (P=0.001). These findings highlight BCAA supplementation as a promising nutritional strategy to mitigate the adverse effects of protein restriction, thereby supporting broiler growth through coordinated improvements in skeletal health, intestinal structure, and nutrient utilization at the molecular level.

## Introduction

Chicken meat is recognized globally as an economical and high-quality source of animal protein (Uzundumlu and Dilli, 2022). With the continuous growth of the human population, the demand for poultry meat has steadily increased, driving rapid expansion of the poultry sector and a marked rise in global production over the last decade (Barbut and Leishman, 2022). Despite this growth, nutrient excretion—particularly nitrogen—remains a critical challenge in poultry production due to its adverse environmental effects (Oluwabiyi and Song, 2024). Consequently, low crude protein (**CP**) diets have been introduced as a nutritional approach to reduce nitrogen output (Selle et al., 2023). Since protein is among the most expensive feed ingredients, reducing dietary CP also helps lower broiler production expenses. However, the use of low-CP diets frequently results in poor skeletal development and altered intestinal morphology, ultimately compromising growth performance. To mitigate these drawbacks, branched-chain amino acids (**BCAA**) comprising isoleucine, leucine, and valine have attracted attention. These essential amino acids cannot be synthesized endogenously in animals and must be supplied through the diet (Oluwabiyi and Song, 2024). BCAA possess structurally similar branched R groups, a feature that defines their name, and they share common catabolic pathways (de Oliveira et al., 2025). As functional amino acids, BCAA play a significant metabolic and regulatory role in the body and account for one-third of the essential amino acids in muscle protein (Kim et al., 2022). BCAA serve not only as building blocks for protein accretion but also as signaling molecules that activate the mTOR pathway, promoting cellular proliferation and differentiation (Jiang et al., 2023). Previous studies have highlighted their potential to enhance muscle growth, support immune function, and improve feed efficiency, yet the underlying mechanisms by which BCAA modulate bone and intestinal development remain insufficiently explored.

Optimal skeletal and intestinal development are critical determinants of growth performance and overall health in broiler chickens (Biesek et al., 2022). Modern broiler strains have been selected for rapid growth and high feed efficiency, which imposes considerable physiological demands on the musculoskeletal system. This accelerated growth often leads to skeletal weaknesses, including reduced bone strength and increased susceptibility to fractures, which can negatively affect welfare and production efficiency (Pedersen et al., 2020). Concurrently, intestinal development plays a pivotal role in nutrient absorption, providing essential minerals, amino acids, and energy substrates required for both skeletal and systemic growth (Ravindran and Abdollahi, 2021). Impaired intestinal integrity or suboptimal morphology can limit nutrient uptake, thereby compromising bone mineralization and overall growth performance (Thanabalan and Kiarie, 2021). At the molecular level, skeletal development is tightly regulated by a network of genes that control osteoblast differentiation, matrix synthesis, and mineralization (Rolfe et al., 2014). Key osteogenic genes, orchestrate bone formation and remodeling. Emerging evidence suggests that BCAA supplementation can modulate the expression of these genes, thereby enhancing bone strength, mineral density, and overall skeletal integrity (Choi et al., 2024). Intestinal morphology is another critical determinant of nutrient utilization and overall growth. Tight junction proteins, including *CLDN-1, OCLN, ZO-1*, and the mucin *MUC-2*, play essential roles in maintaining epithelial barrier integrity, regulating paracellular permeability, and protecting the mucosal surface from luminal insults (Zeisel et al., 2019). Enhanced expression of these genes has been associated with improved villus height, crypt depth, and absorptive surface area, which in turn facilitate the uptake of minerals, amino acids, and other nutrients critical for skeletal development (Sivri et al., 2025). Finally, the absorption of BCAA and other essential amino acids is mediated by specific intestinal transporters, primarily members of the solute carrier (**SLC**) family (Liang et al., 2024). These transporters facilitate the uptake of neutral and cationic amino acids into enterocytes, directly influencing systemic amino acid availability, protein synthesis, and growth. Upregulation of these transporters may enhance amino acid absorption efficiency, thereby supporting both intestinal development and skeletal growth (Wang et al., 2020).

Despite extensive research on the effects of BCAA on growth performance and muscle development, there is limited information on their simultaneous impact on skeletal quality, intestinal morphology, and the expression of genes involved in bone metabolism, intestinal integrity, and amino acid transport in broilers. Therefore, the present study aimed to investigate the effects of dietary BCAA supplementation in low CP on growth performance, bone quality parameters, intestinal morphometric traits, and the expression of key genes associated with skeletal development, intestinal barrier function, and amino acid transport in broiler chickens. By integrating these aspects, we sought to provide a mechanistic understanding of how BCAA influence growth and development, highlighting their potential as a nutritional strategy to optimize skeletal and intestinal health in modern broiler production systems.

## Materials and methods

All of the animal experimentation procedures used in the experiment were approved by the Animal Care Committee and Animal Research Ethics Board, Islamic Azad University- Central Tehran Branch, Iran (Approval No: IR.IAU.CTB.REC.1402.127) and were in compliance with the European Union guidelines for the care and use of animals in research (European Parliament, 2010).

### Birds, diets, and management

In this study, a total of 480 one‑day‑old male and female broiler chicks (Ross 308 strain) were used. The average body weight of the chicks at day one was 43.62 ± 0.36 g. The experiment was conducted using a completely randomized design consisting of four treatments. Each treatment included six replicates, and 20 chicks were randomly assigned to each replicate (10 males and 10 females). The experimental diets were based on a corn–soybean meal formulation and included the following treatments: 1. a standard protein and BCAA diet (NCP), 2. diet with 10% less protein than the standard and BCAA standard (LCP), 3. LCP diet with 10% more BCAA than the requirement (LCP+10BCAA), and 4. LCP diet with 20% more BCAA than the requirement (LCP+20BCAA). All diets were formulated according to the nutritional recommendations provided in the Ross 308 Nutrition Specifications (2022 edition) and were balanced using UFFDA ration formulation software. The ratio of metabolizable energy to CP in all diets was adjusted based on the recommended standards for the Ross 308 strain. The formulated diets used during the starter (1–10 days), grower (11–24 days), and finisher (25–42 days) phases are presented in Table 1, Table 2, Table 3. Throughout the entire rearing period, feed and drinking water were supplied ad libitum. Management practices including lighting program, ventilation, ambient temperature, stocking density, and litter conditions were kept identical for all treatments and were implemented according to the recommended management guidelines. Five samples from each experimental diet were collected for the determination of gross energy, CP, calcium, phosphorus, and amino acid concentrations. Experimental diets were ground to pass through a 0.75-mm sieve using a laboratory mill grinder (ZM 100; Retsch GmbH, Haan, Germany). Diet samples were then ashed in a muffle furnace at 600°C for 16 h for subsequent determination of calcium and phosphorus. The concentrations of calcium and phosphorus in the samples were measured using flame atomic absorption spectrophotometry (Varian SpectrAA 50B Atomic Absorption Spectrometer; Varian Ltd., USA) and a spectrophotometer (Jenway Genova MK3, UK), respectively. The gross energy of the experimental diets was measured using a bomb calorimeter (Parr 6200; Parr Instruments Co., Moline, IL, USA), with benzoic acid used as the calibration standard. CP content was determined by measuring nitrogen concentration using the micro-Kjeldahl method and multiplying the obtained value by 6.25. In addition, amino acid concentrations were quantified using an automatic amino acid analyzer (Hitachi L-8800, Tokyo, Japan) equipped with a ninhydrin reagent system, lithium buffer system, and photometric detection for individual amino acids.

**Table 1:** Components and chemical compositions of diets used in starter period (DM basis).

Ingredients, %	Standard	Low-CP	LCP+10BCAA	LCP+20BCAA
Maize	60.70	63.37	62.54	61.78
Soybean meal (44% CP)	27.58	26.84	26.24	25.79
Corn gluten	6.69	4.57	5.85	6.90
Calcium carbonate	1.70	1.70	1.70	1.70
Dicalcium phosphate	1.54	1.52	1.52	1.52
Salt	0.30	0.30	0.30	0.30
Vitamin-mineral mix*	0.50	0.50	0.50	0.50
Sodium bicarbonate	0.07	0.07	0.07	0.07
Lysine-HCL	0.57	0.60	0.61	0.62
DL-methionine	0.17	0.22	0.22	0.22
L-threonine	0.07	0.11	0.11	0.11
L-isoleucine	0.01	0.03	0.08	0.12
L-valine	-	0.02	0.09	0.18
L-arginine	0.10	0.15	0.17	0.19
**Nutrient levels**				
Metabolizable energy, Kcal/kg	2950	2950	2950	2950
Crude protein, %	22.23	20.01	20.01	20.01
Calcium, %	0.93	0.93	0.93	0.93
Available phosphorus, %	0.46	0.46	0.46	0.46
Dig. Lysine, %	1.40	1.40	1.40	1.40
Dig. Methionine, %	0.55	0.56	0.56	0.56
Dig. Met + Cyst, %	0.92	0.92	0.92	0.92
Dig. Threonine, %	0.83	0.83	0.83	0.83
Dig. Arginine, %	1.32	1.32	1.32	1.32
Dig. Valine, %	1.00	1.00	1.10	1.20
Dig. Leucine, %	1.31	1.30	1.43	1.56
Dig. Isoleucine, %	0.89	0.89	0.98	1.06
**Analyzed value**				
Gross energy, Kcal/kg	4241	4237	4256	4244
Crude protein, %	22.25	20.02	20.00	20.04
Calcium, %	0.92	0.93	0.94	0.93
Total phosphorus, %	0.72	0.71	0.73	0.72
Lysine, %	1.54	1.56	1.55	1.54
Methionine, %	0.61	0.62	0.61	0.62
Met + Cyst, %	0.98	1.01	1.02	0.99
Threonine, %	0.95	0.94	0.95	0.96
Arginine, %	1.45	1.45	1.46	1.44
Valine, %	1.16	1.15	1.28	1.40
Leucine, %	1.38	1.39	1.51	1.65
Isoleucine, %	0.97	0.97	1.07	1.16

⁎ Supplied per kilogram of diet: vitamin A, 9000 U; vitamin D3, 2000 U; vitamin E, 18 U; vitamin B12, 0.15 mg; riboflavin, 6.6 mg; calcium pantothenate, 10 mg; niacin, 30 mg; choline, 500 mg; biotin, 0.1 mg; thiamine, 1.8 mg; Pyridoxin, 3 mg; folic acid, 1 mg; vitamin K3., 2 mg; antioxidant (Ethoxyquin), 100 mg; zinc, 50 mg; manganese oxide, 100 mg; copper, 10 mg; Fe, 50 mg; I, 1 mg; Se, 0.2 mg.

**Table 2:** Components and chemical compositions of diets used in grower period (DM basis).

Ingredients, %	Standard	Low-CP	LCP+10BCAA	LCP+20BCAA
Maize	55.45	61.72	59.87	59.09
Soybean meal (44% CP)	36.38	31.07	30.21	28.12
Corn gluten	-	-	1.81	3.67
Soybean oil	3.92	2.60	3.43	4.14
Calcium carbonate	1.53	1.54	1.54	1.54
Dicalcium phosphate	1.30	1.33	1.33	1.33
Salt	0.30	0.30	0.30	0.30
Vitamin-mineral mix*	0.50	0.50	0.50	0.50
Sodium bicarbonate	0.08	0.07	0.07	0.07
Lysine-HCL	0.33	0.38	0.39	0.46
DL-methionine	0.19	0.23	0.23	0.22
L-threonine	-	0.04	0.05	0.06
L-valine	0.01	0.06	0.11	0.21
L-isoleucine	0.01	0.08	0.09	0.17
L-arginine	-	0.08	0.07	0.12
**Nutrient levels**				
Metabolizable energy, Kcal/kg	3050	3050	3050	3050
Crude protein, %	21.15	19.04	19.04	19.04
Calcium, %	0.85	0.85	0.85	0.85
Available phosphorus, %	0.43	0.43	0.43	0.43
Dig. Lysine, %	1.39	1.39	1.39	1.39
Dig. Methionine, %	0.52	0.52	0.52	0.52
Dig. Met + Cyst, %	0.86	0.86	0.86	0.86
Dig. Threonine, %	0.79	0.76	0.76	0.76
Dig. Arginine, %	1.35	1.34	1.34	1.34
Dig. Valine, %	0.98	0.98	1.08	1.18
Dig. Leucine, %	1.19	1.19	1.30	1.42
Dig. Isoleucine, %	0.87	0.87	0.96	1.04
**Analyzed value**				
Gross energy, Kcal/kg	4357	4368	4351	4365
Crude protein, %	21.10	19.01	19.03	19.00
Calcium, %	0.83	0.85	0.84	0.83
Total phosphorus, %	0.68	0.69	0.68	0.67
Lysine, %	1.47	1.47	1.46	1.48
Methionine, %	0.57	0.57	0.55	0.56
Met + Cyst, %	0.93	0.94	0.93	0.94
Threonine, %	0.94	0.95	0.93	0.95
Arginine, %	1.47	1.46	1.48	1.47
Valine, %	1.13	1.15	1.24	1.35
Leucine, %	1.27	1.27	1.40	1.52
Isoleucine, %	0.95	0.96	1.04	1.15

⁎ Supplied per kilogram of diet: vitamin A, 9000 U; vitamin D3, 2000 U; vitamin E, 18 U; vitamin B12, 0.15 mg; riboflavin, 6.6 mg; calcium pantothenate, 10 mg; niacin, 30 mg; choline, 500 mg; biotin, 0.1 mg; thiamine, 1.8 mg; Pyridoxin, 3 mg; folic acid, 1 mg; vitamin K3., 2 mg; antioxidant (Ethoxyquin), 100 mg; zinc, 50 mg; manganese oxide, 100 mg; copper, 10 mg; Fe, 50 mg; I, 1 mg; Se, 0.2 mg.

**Table 3:** Components and chemical compositions of diets used in finisher period (DM basis).

Ingredients, %	Standard	Low-CP	LCP+10BCAA	LCP+20BCAA
Maize	60.55	67.68	67.62	67.35
Soybean meal (44% CP)	31.00	24.83	22.23	20.28
Corn gluten	-	-	2.00	3.50
Soybean oil	4.01	3.28	3.63	4.05
Calcium carbonate	1.40	1.42	1.42	1.42
Dicalcium phosphate	1.68	1.21	1.21	1.21
Salt	0.30	0.30	0.30	0.30
Vitamin-mineral mix*	0.50	0.50	0.50	0.50
Sodium bicarbonate	0.07	0.07	0.07	0.07
Lysine-HCL	0.31	0.35	0.40	0.47
DL-methionine	0.17	0.22	0.22	0.21
L-threonine	-	0.03	0.05	0.07
L-valine	-	0.05	0.15	0.24
L-isoleucine	0.01	0.02	0.09	0.16
L-arginine	-	0.04	0.11	0.17
**Nutrient levels**				
Metabolizable energy, Kcal/kg	3150	3150	3150	3150
Crude protein, %	19.20	17.28	17.28	17.28
Calcium, %	0.86	0.86	0.86	0.86
Available phosphorus, %	0.39	0.39	0.39	0.39
Dig. Lysine, %	1.25	1.25	1.25	1.25
Dig. Methionine, %	0.47	0.47	0.47	0.47
Dig. Met + Cyst, %	0.79	0.79	0.79	0.79
Dig. Threonine, %	0.71	0.71	0.71	0.71
Dig. Arginine, %	1.20	1.20	1.20	1.20
Dig. Valine, %	0.88	0.88	0.97	1.06
Dig. Leucine, %	1.26	1.26	1.39	1.51
Dig. Isoleucine, %	0.80	0.80	0.88	0.96
**Analyzed value**				
Gross energy, Kcal/kg	4473	4481	4465	4479
Crude protein, %	19.17	17.30	17.31	17.27
Calcium, %	0.84	0.83	0.85	0.84
Total phosphorus, %	0.61	0.63	0.62	0.61
Lysine, %	1.33	1.33	1.32	1.31
Methionine, %	0.54	0.56	0.55	0.55
Met + Cyst, %	0.86	0.85	0.86	0.87
Threonine, %	0.81	0.82	0.81	0.83
Arginine, %	1.28	1.30	1.29	1.30
Valine, %	1.02	1.00	1.12	1.23
Leucine, %	1.35	1.36	1.48	1.63
Isoleucine, %	0.86	0.86	0.95	1.04

⁎ Supplied per kilogram of diet: vitamin A, 9000 U; vitamin D3, 2000 U; vitamin E, 18 U; vitamin B12, 0.15 mg; riboflavin, 6.6 mg; calcium pantothenate, 10 mg; niacin, 30 mg; choline, 500 mg; biotin, 0.1 mg; thiamine, 1.8 mg; Pyridoxin, 3 mg; folic acid, 1 mg; vitamin K3., 2 mg; antioxidant (Ethoxyquin), 100 mg; zinc, 50 mg; manganese oxide, 100 mg; copper, 10 mg; Fe, 50 mg; I, 1 mg; Se, 0.2 mg.

### Performance

Feed intake (**FI**) was recorded daily after weighing the feed provided to the birds. To determine the FI for each replicate, the amount of feed remaining at the end of each rearing period was subtracted from the total amount of feed offered during that period. Body weight gain (**BWG**) for each replicate in each experimental period was calculated by determining the difference between the final body weight and the body weight at the beginning of that period. On days 1, 10, 24, and 42 of age, all birds in each experimental unit were weighed collectively to determine body weight. The feed conversion ratio (**FCR**) was calculated separately for the starter, grower, and finisher phases. This parameter was obtained by dividing the average feed intake by the average body weight gain of the birds within each period. During the experimental period, mortality was monitored daily in each pen before feed distribution, and the weight of the dead birds was recorded. The daily mortality data were used to calculate the chicken‑day value for each experimental unit (Ebrahimi et al., 2026).

### Bone quality

At the end of the experimental period, two male birds from each replicate were randomly selected and slaughtered. Subsequently, the tibia bones were collected. After removing all surrounding tissues, the left tibia from each slaughtered bird was transferred to a freezer for further analyses. The weight of each tibia bone was determined using a digital balance with a precision of 0.01 g (KEB 602, China). The resistance of the left tibia to pressure was measured and expressed in newtons (Pedersen et al., 2020). This mechanical test was performed using a Santam testing machine (model DBBP‑500, Iran) at a loading speed of 60 mm/min. For all samples, the pressure was applied at the same anatomical location, and the midpoint of the bone shaft was selected as the loading point. The ultimate breaking force of the tibia, defined as the first indication of fracture, was obtained directly from the load–deformation curve recorded by a computerized monitoring system (Liu et al., 2017). Following the mechanical strength test, the tibia samples were transferred to an electric furnace and heated at 600 °C for 16 hours in order to determine bone ash content (AOAC, 2006). After removal from the furnace and cooling to room temperature, the remaining ash was weighed, and the ash percentage for each sample was calculated. The calcium concentration in tibia samples was determined using flame atomic absorption spectrophotometry (Varian Spectr AA 50B Atomic Absorption Spectrometer; Varian Ltd., USA) according to AOAC (2006). In addition, tibia phosphorus content was measured using a spectrophotometer (Jenway Genova MK3, UK) following the procedures described by AOAC (2006).

### Intestinal morphology

At 42 days of age, two male birds from each replicate (12 birds in total) were selected, and a segment of the jejunum was collected for morphological assessment following the procedure described by Khalilzadeh et al. (2025). Approximately 1 cm of the jejunum was excised and gently rinsed with 0.9% saline solution to remove any intestinal contents. The collected tissue samples were then fixed in 10% buffered formalin to preserve their structure for subsequent histological examination. Following fixation, the samples underwent routine histological processing, including dehydration, clearing, and embedding in paraffin. The paraffin-embedded tissues were then sectioned at a thickness of 7 μm using a microtome (Microm, HM 335), and the resulting sections were mounted on glass slides. The prepared slides were first deparaffinized using xylene and subsequently rehydrated through a graded series of ethanol solutions. Afterward, the tissue sections were stained with hematoxylin and eosin (H&E) and examined using a light microscope. Morphological parameters evaluated in the intestinal samples included villus height, villus width, crypt depth, villus surface area, thickness of the muscularis layer, and the number of goblet cells. Villus height was determined by measuring the distance from the villus tip to the upper boundary of the lamina propria. Crypt depth was measured from the base of the villus-crypt junction to the tip of the muscularis mucosa. The ratio of villus height to crypt depth (VH/CD) was calculated by dividing villus height by crypt depth (Hassanlou et al., 2026). Additionally, the surface area of the villi was estimated using the formula (2π) × (villus width × 0.5) × villus length (Sakamoto et al., 2000).

### Sample collection

For the gene expression analysis, the right tibia was excised from broiler chickens (12 male birds from each treatment group), and after complete removal of surrounding soft tissues, the diaphyseal portion was collected as the bone sample. In addition, a ∼2 cm segment from the mid-jejunum was obtained; its contents were gently flushed with ice-cold PBS, and the mucosal layer was scraped using a sterile glass slide. All collected samples (n = 12 per treatment, with two samples taken from each pen) were immediately frozen after collection and stored at −80°C until RNA extraction was performed (Ghaleghafi et al., 2025).

### Quantitative real-time PCR

Total RNA was extracted from approximately 30 mg of tissue using RNAiso Plus reagent (TaKaRa) following the manufacturer’s protocol. The concentration and purity of the isolated RNA were evaluated using a spectrophotometer (NanoDrop-1000; Thermo Fisher Scientific). Subsequently, complementary DNA (cDNA) was synthesized from the extracted total RNA through reverse transcription using the PrimeScript RT reagent kit (Takara). Quantitative real-time PCR (qRT-PCR) analysis was carried out using SYBR Green Master Mix, and each sample was analyzed in duplicate with a StepOne real-time thermocycler (Applied Biosystems, Foster City, CA, USA). The amplification program for all target genes consisted of an initial denaturation step at 95 °C for 10 minutes, followed by 40 amplification cycles including denaturation at 95 °C for 15 seconds, annealing at the specific primer annealing temperature for 20 seconds, and an extension step at 72 °C for 15 seconds. To normalize gene expression data, the geometric mean of two housekeeping genes, glyceraldehyde-3-phosphate dehydrogenase (GAPDH) and β-actin, was used as the internal reference. Relative expression levels of the target genes were then calculated according to the 2^−ΔΔCt^ method described by Livak and Schmittgen (2001). The sequences of primers used for both housekeeping and target genes are presented in Table 4.

**Table 4:** Primers used in gene quantification by qPCR.

Gene	Accession No.	Forward primer	Reverse primer
*β-Actin*	NM_205518.1	GAGAAATTGTGCGTGACATCA	CCTGAACCTCTCATTGCCA
*GAPDH*	NM_204305.1	CTTTGGCATTGTGGAGGGTC	ACGCTGGGATGATGTTCTGG
***Bone metabolism-related enzyme and proteins***			
*ALP*	NM_205360.1	GGAGAAGGACCCCGAATACTG	TTGACGCCGCAGAGGTAAG
*RUNX-2*	NM_204474.2	TTCACAAGCATTTCATCCCTC	TTGCGGACATACCCAGTGACA
*FGF-23*	XM425663.2	ATGCTGCTTGTGCTCTGTATC	ACTGTAAATGGTTTGGTGAGG
*BGLAP*	NM_205387.3	TGCTCGCAGTGCTAAAGCCTTCAT	TCAGCTCACACACCTCTCGTT
*COL-1*	NM_205098.1	GTCATTCCACCCCACGTCAT	CACCACTGTCACCTCTCAGAC
*RANKL*	XM_021276016.1	CATGGATCTCCCGATGAGCA	CTACTAGGGTCCATCTCAGTCC
*BMP-2*	NM_205513.1	AGCTTCCACCACGAAGAAGT	GGTAGCTGCTGTTGCTCTCA
***Tight junction proteins***			
*CLDN-1*	NM_001013611.2	TGGAGGATGACCAGGTGAAGA	CGAGCCACTCTGTTGCCATA
*CLDN-2*	NM_001277622.1	CCTGCTCACCCTCATTGGAG	GCTGAACTCACTCTTGGGCT
*OCLN*	XM_025144248.1	ACGGCAGCACCTACCTCAA	GGCGAAGAAGCAGATGAG
*ZO-1*	XM_015278981.2	CAACTGGTGTGGGTTTCTGAA	TCACTACCAGGAGCTGAGAGGTAA
*ZO-2*	NM_204918.1	ATCCAAGAAGGCACCTCAGC	CATCCTCCCGAACAATGC
***Mucin***			
*MUC-2*	JX_284122.1	ATGCGATGTTAACACAGGACTC	GTGGAGCACAGCAGACTTTG
***Nutrient transporter***			
*SLC3A1*	XM_040667709.2	GGCTTACCAATGGAGCTGAGTGAG	CTTGGCTGCTGGTGTCAGTATCC
*SLC6A14*	XM_040670974.2	CTCCAGTGGGCTGGATGAGA	CAAGGCAGCTCCAACGATCA
*SLC1A1*	NM_001298770.1	CCTGTCACCTTCCGCTGTGC	GCTGCTGTTGCTGTGACACTAATG
*SLC7A1*	XM_046908303.1	GGAGCCTTGTGGGTGTGTAA	CTAGGGGAACGCTGCTAAGT
*SLC7A6*	XM_046925772.1	GAAGCAGAACGAGGAGCCAT	TGGTTGCAGCTGTGAGGAAT
*SLC7AL*	XM_046911929.1	CAGAAAACCTCAGAGCTCCCTTT	TGAGTACAGAGCCAGCGCAAT

Genes associated with bone metabolism that were evaluated in this study included alkaline phosphatase (*ALP*), runt-related transcription factor 2 (*RUNX-2*), fibroblast growth factor 23 (*FGF-23*), bone γ-carboxyglutamate protein (*BGLAP*), collagen type I alpha 1 chain (*COL-1*), receptor activator of nuclear factor κB ligand (*RANKL*), and bone morphogenetic protein 2 (*BMP-2*). To assess intestinal barrier integrity, several tight junction proteins and mucin-related genes were analyzed, including claudin-1 (*CLDN-1*), claudin-2 (*CLDN-2*), occludin (*OCLN*), zonula occludens-1 (*ZO-1*), zonula occludens-2 (*ZO-2*), and mucin-2 (*MUC-2*). Furthermore, genes involved in nutrient transport were also examined. These included solute carrier family 3 member 1 (*SLC3A1*), solute carrier family 6 member 14 (*SLC6A14*), solute carrier family 1 member 1 (*SLC1A1*), solute carrier family 7 member 1 (*SLC7A1*), solute carrier family 7 member 6 (*SLC7A6*), and solute carrier family 7-like (*SLC7AL*).

### Statistical analysis

All data were analyzed using the general linear model's ANOVA function in SAS software (version 9.4; SAS Institute Inc., Cary, NC, USA). For performance variables, the statistical model used was *Y_ij_* =*μ* +*T_i_* +*e_ij_*​. For the remaining variables, the model was *Y_ijk_* = *μ* + *T_i_* + *e_ij_* + *ε_ijk_*. In these models, *Y_ij_*​ and *Y_ijk_*​ denote the observed responses, *μ* represents the overall mean, *Ti*​ corresponds to the dietary treatment effect, *e_ij_*​ is the random error term, and *ε_ijk_* refers to the subsampling error. For growth performance traits, the pen was considered the experimental unit, whereas the individual bird was considered the experimental unit for bone quality parameters, intestinal morphology, and gene expression. Before performing analysis of variance, data distribution was evaluated for normality using the Shapiro–Wilk, Kolmogorov–Smirnov, Cramér–von Mises, and Anderson–Darling tests within the UNIVARIATE procedure of SAS. Homogeneity of variances was also evaluated using Levene’s, Brown–Forsythe, and Bartlett’s tests. Mean comparisons were performed using Tukey’s multiple range test with a significance level of *P <0.05*.

## Results

### Broiler performance

**Fig. 1:**
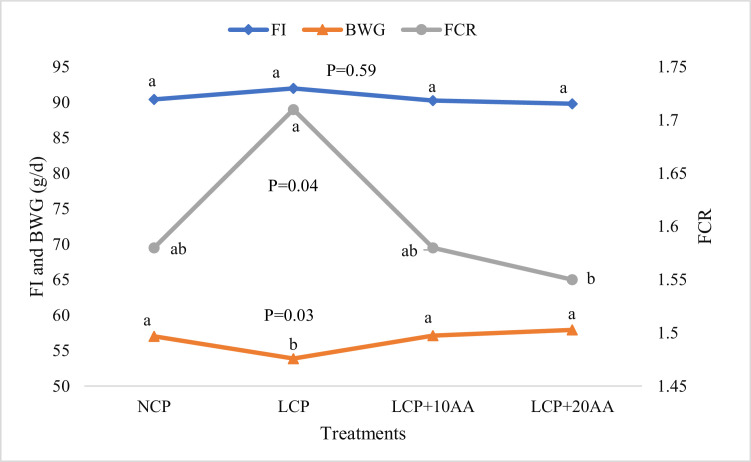
The effect of branched-chain amino acids in low-protein diets on the performance of broiler chickens in total period (1-42d). Experimental diets included a standard protein diet (NCP), a diet with 10% less protein than the standard (LCP), an LCP diet with 10% more BCAA than the requirement (LCP+10BCAA), and an LCP diet with 20% more BCAA than the requirement (LCP+20BCAA). Different letters (a, b) indicate significant differences at P < 0.05.

The effects of BCAAs in low-protein diets on the performance of broiler chickens during the total period are presented in Fig. 1. FI was not significantly affected by dietary treatments (P = 0.59). BWG was significantly higher in the NCP, LCP+10BCAA, and LCP+20BCAA groups compared to the LCP group (P = 0.03). The FCR was significantly improved in the LCP+20BCAA group compared with the LCP group (P = 0.04).

### Bone quality

**Fig. 2:**
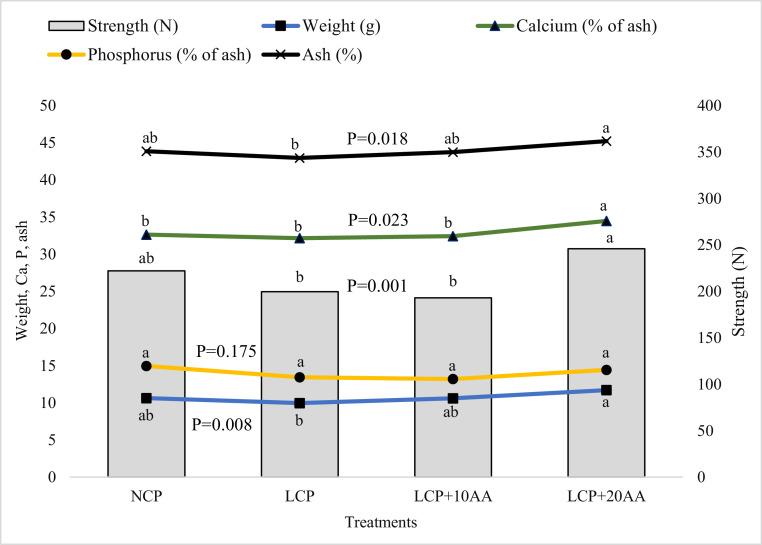
The effect of branched-chain amino acids in low-protein diets on tibia bone quality of broilers at the age of 42 days (n=12). Experimental diets included a standard protein diet (NCP), a diet with 10% less protein than the standard (LCP), an LCP diet with 10% more BCAA than the requirement (LCP+10BCAA), and an LCP diet with 20% more BCAA than the requirement (LCP+20BCAA). Different letters (a, b) indicate significant differences at P < 0.05.

Fig. 2 presents the results of tibia bone quality. Tibia weight and Ash was improved in the LCP+20BCAA group compared with LCP (P < 0.05). Bone strength reached its highest value in the LCP+20BCAA group, which differed significantly from both LCP and LCP+10BCAA (P = 0.001). Similarly, tibia calcium concentration was elevated in the LCP+20BCAA group in comparison with all other treatments (P = 0.023). No treatment effect was detected for tibia phosphorus (P = 0.175).

### Jejunum morphology

The effect of BCAAs in low-protein diets on the morphology of the jejunum of broiler chickens is reported in Table 5. Villus height was significantly reduced in the LCP group compared with NCP, while supplementation with BCAA restored and further enhanced villus height, with the highest values observed in the LCP + 20BCAA group (P = 0.009). A similar pattern was observed for villus surface area, where the LCP group showed the lowest value, but BCAA supplementation significantly increased surface area, particularly in the LCP + 20BCAA group (P = 0.001), indicating improved absorptive capacity. In contrast, villus width and crypt depth did not differ significantly among treatments (P = 0.094 and P = 0.276, respectively). However, the muscularis layer thickness was markedly higher in both BCAA-supplemented groups compared to NCP and LCP (P = 0.001). Furthermore, the number of goblet cells was significantly increased in the LCP + 20BCAA group compared with LCP (P = 0.021). The villus height-to-crypt depth ratio (VH/CD) was significantly higher in NCP, LCP + 10BCAA, and LCP + 20BCAA groups compared to LCP (P = 0.001), suggesting that BCAA supplementation alleviated the negative effects of low-protein diets on intestinal development.

**Table 5:** The effect of branched-chain amino acids in low-protein diets on jejunum morphology of broilers at d 42 (n=12).

Items	NCP	LCP	LCP+10BCAA	LCP+20BCAA	SEM	P-value
Villus height (μm)	1087.16^b^	982.64^c^	1100.28^b^	1207.67^a^	18.05	0.009
Villus width (μm)	179.62	178.84	192.65	195.52	6.80	0.094
Crypt depth (μm)	166.37	177.64	171.81	175.29	5.18	0.276
Villus surface area (µm²)	610959.3^b^	551942.9^c^	663168.5^b^	739046.2^a^	1812.29	0.001
Muscularis layer (μm)	173.35^b^	171.06^b^	203.11^a^	199.34^a^	5.63	0.001
Goblet cells (n)	7532.37^ab^	7265.61^b^	7753.27^ab^	7868.64^a^	84.37	0.021
VH/ CD	6.54^a^	5.53^b^	6.42^a^	6.89^a^	0.21	0.001

Experimental diets included a standard protein diet (NCP), a diet with 10% less protein than the standard (LCP), an LCP diet with 10% more BCAA than the requirement (LCP+10BCAA), and an LCP diet with 20% more BCAA than the requirement (LCP+20BCAA). SEM: standard error of means.

a-b Means within a variable with no common superscript differ significantly (*p*< 0.05).

### Bone metabolism-related genes

The effects of BCAAs on the mRNA expression of bone metabolism-related genes in broilers are summarized in Table 6. Among the genes examined, *RUNX-2, BGLAP, BMP-2, ALP*, and *COL-1* exhibited significant responses to dietary BCAA supplementation (P < 0.05). Specifically, *RUNX-2* expression was markedly upregulated in both LCP + 10BCAA and LCP + 20BCAA groups compared to LCP and NCP, indicating a strong stimulatory effect of BCAA on osteogenic transcriptional activity (P = 0.001). Similarly, *BGLAP* and *BMP-2* showed consistent significant increases in the supplemented groups, suggesting enhanced osteoblast differentiation and bone matrix formation (P < 0.05). *ALP* and *COL-1* also increased in diets containing BCAA supplementation and were significantly different from the NCP group (P < 0.05). In contrast, *RANKL* did not show significant changes among the treatments (P = 0.219), indicating that BCAA supplementation had minimal influence on osteoclastogenesis signaling in this experiment. *FGF-23* expression was significantly elevated only in the LCP + 20BCAA group compared to LCP and NCP (P = 0.002).

**Table 6:** Effect of branched-chain amino acids in low-protein diets on the mRNA expression of bone metabolism-related genes at d 42 (n=12).

Gene	NCP	LCP	LCP+10BCAA	LCP+20BCAA	SEM	P-value
*ALP*	1.000^ab^	0.903^b^	1.087^a^	1.274^a^	0.031	0.016
*RUNX-2*	1.000^b^	0.885^c^	1.332^a^	1.326^a^	0.022	0.001
*FGF-23*	1.000^b^	0.952^b^	0.981^b^	1.129^a^	0.037	0.002
*BGLAP*	1.000^b^	0.824^c^	1.082^b^	1.304^a^	0.030	0.001
*COL-1*	1.000^ab^	0.917^b^	1.056^a^	1.261^a^	0.045	0.025
*RANKL*	1.000	0.983	1.042	1.098	0.052	0.219
*BMP-2*	1.000^b^	0.948^b^	1.217^a^	1.185^a^	0.026	0.009

Experimental diets included a standard protein diet (NCP), a diet with 10% less protein than the standard (LCP), an LCP diet with 10% more BCAA than the requirement (LCP+10BCAA), and an LCP diet with 20% more BCAA than the requirement (LCP+20BCAA).

Abbreviations: ALP: alkaline phosphatase; RUNX-2: runt-related transcription factor 2; FGF-23: fibroblast growth factor 23; BGLAP: bone γ-carboxyglutamate protein; COL-1: collagen type I alpha 1 chain; RANKL: receptor activator of nuclear factor κB ligand; BMP-2: bone morphogenetic protein 2; SEM: standard error of means.

a-b Means within a variable with no common superscript differ significantly (*p*< 0.05).

### Tight junction proteins and mucin-related genes

The expression of tight junction proteins and mucin-related genes in the jejunum mucosa showed distinct responses to dietary BCAA supplementation (Table 7). *CLDN-1* expression significantly decreased in the LCP group compared to NCP, while supplementation with BCAA restored and further upregulated its expression, with the highest level observed in the LCP + 20BCAA group (P = 0.026). A similar pattern was observed for *OCLN*, where the LCP group showed downregulation, but supplementation with 20% BCAA markedly enhanced its expression compared to all other groups (P = 0.007). *ZO-1* demonstrated the strongest response, with a significant increase in both LCP + 10BCAA and LCP + 20BCAA groups relative to NCP and LCP (P = 0.001). In contrast, *CLDN-2* and *ZO-2* expression levels did not differ significantly among treatments, suggesting that these genes are less responsive to dietary BCAA under the current conditions (P > 0.05). Finally, *MUC-2*, a key marker of goblet cell activity and mucosal protection, was significantly upregulated in BCAA-supplemented groups compared with NCP and LCP (P = 0.001).

**Table 7:** Effect of branched-chain amino acids in low-protein diets on gene expression of tight junction proteins and mucin in the jejunum mucosal at d 42 (n=12).

Gene	NCP	LCP	LCP+10BCAA	LCP+20BCAA	SEM	P-value
*CLDN-1*	1.000^b^	0.854^c^	1.168^ab^	1.352^a^	0.061	0.026
*CLDN-2*	1.000	0.967	1.049	1.035	0.043	0.391
*OCLN*	1.000^b^	0.746^c^	0.950^b^	1.261^a^	0.031	0.007
*ZO-1*	1.000^b^	0.947^b^	1.187^a^	1.395^a^	0.050	0.001
*ZO-2*	1.000	0.930	1.066	0.973	0.042	0.180
*MUC-2*	1.000^b^	0.889^c^	1.244^a^	1.292^a^	0.037	0.001

Experimental diets included a standard protein diet (NCP), a diet with 10% less protein than the standard (LCP), an LCP diet with 10% more BCAA than the requirement (LCP+10BCAA), and an LCP diet with 20% more BCAA than the requirement (LCP+20BCAA).

Abbreviations: CLDN-1: claudin-1; CLDN-2: claudin-2; OCLN: occluding; ZO-1: zonula occludens-1: ZO-2: zonula occludens-2; MUC-2: mucin-2; SEM: standard error of means.

a-b Means within a variable with no common superscript differ significantly (*p*< 0.05).

### Nutrient transporter-related genes

The effects of BCAA supplementation on nutrient transporter gene expression in the jejunum are shown in Table 8. Among the genes, *SLC3A1, SLC6A14*, and *SLC7A6* demonstrated significant changes (P < 0.05). *SLC3A1*, which encodes a heavy subunit of amino acid transporters, was markedly reduced in the LCP group but significantly increased in the LCP + 20BCAA treatment (P = 0.001). Similarly, *SLC6A14* expression was enhanced in both BCAA-supplemented groups compared with LCP (P = 0.035), suggesting improved transport of cationic and neutral amino acids under BCAA supplementation. The most prominent effect was observed for *SLC7A6*, where expression levels dropped sharply in the LCP and LCP + 10BCAA groups but increased dramatically in the LCP + 20BCAA treatment (P = 0.001), pointing to a dose-dependent response. In contrast, *SLC1A1, SLC7A1*, and *SLC7AL* did not show significant differences among treatments (P = 0.108, 0.215, and 0.298, respectively).

**Table 8:** Effect of branched-chain amino acids in low-protein diets on the mRNA expression of nutrient transporter-related genes at d 42 (n=12).

Gene	NCP	LCP	LCP+10BCAA	LCP+20BCAA	SEM	P-value
*SLC3A1*	1.000^b^	0.752^d^	0.985^c^	1.192^a^	0.042	0.002
*SLC6A14*	1.000^ab^	0.826^b^	1.241^a^	1.185^a^	0.069	0.035
*SLC1A1*	1.000	0.964	0.932	0.986	0.048	0.108
*SLC7A1*	1.000	0.907	0.970	1.025	0.052	0.215
*SLC7A6*	1.000^b^	0.813^c^	0.847^c^	1.291^a^	0.037	0.001
*SLC7AL*	1.000	0.974	1.038	1.017	0.051	0.298

Experimental diets included a standard protein diet (NCP), a diet with 10% less protein than the standard (LCP), an LCP diet with 10% more BCAA than the requirement (LCP+10BCAA), and an LCP diet with 20% more BCAA than the requirement (LCP+20BCAA).

Abbreviations: *SLC3A1*: solute carrier family 3 member 1; *SLC6A14*: solute carrier family 6 member 14; *SLC1A1*: solute carrier family 1 member 1; *SLC7A1*: solute carrier family 7 member 1; *SLC7A6*: solute carrier family 7 member 6; *SLC7AL*: solute carrier family 7-like; SEM: standard error of means.

a-b Means within a variable with no common superscript differ significantly (*p*< 0.05).

## Discussion

Our results indicated that broilers fed the LCP exhibited reduced overall growth performance, as shown by lower BWG compared with the NCP. Supplementation with BCAAs at 10% and 20% above the requirement effectively restored growth, with the 20% group achieving the highest weight gain, demonstrating the compensatory potential of BCAAs under protein-restricted conditions. These findings are in line with previous studies; for instance, Oliveira et al. (2023) and Oluwabiyi and Song (2024) reported that BCAA supplementation in low-protein diets improved BWG and feed efficiency in broilers by providing essential substrates for protein synthesis and supporting metabolic demands. Mechanistically, BCAAs especially leucine, serve as direct substrates for muscle protein synthesis and activate the mTOR signaling pathway, which regulates anabolic processes in skeletal muscle (Yu et al., 2024). Additionally, BCAAs increase the availability of amino acids in circulation by upregulating intestinal amino acid transporters (e.g., *SLC3A1, SLC6A14, SLC7A6*), facilitating efficient nutrient absorption (Fotiadis et al., 2013; Table 6). Also, the observed improvements in jejunum morphology, including increased villus height, surface area, and VH/CD ratio (Table 3), likely amplified the absorptive capacity of the intestine, allowing for better utilization of dietary nutrients. These synergistic effects between molecular signaling, intestinal development, and nutrient transport provide a physiological explanation for the enhanced growth performance in BCAA-supplemented broilers under low-protein conditions.

The tibia and femur are the primary weight-bearing bones in broilers. The rapid growth in broilers, from 40g at hatching to 2500g at 6 wk of age, places a considerable strain on their skeletal system (Yair et al., 2012). Studies have indicated that appropriately augmenting mechanical load can improve bone mineral density and strength, thereby positively influencing skeletal adaptation (Uto et al., 2017). However, excessive loading negatively impacts bone growth and development, leading to a deterioration in skeletal structure and mechanical properties (Zhang et al., 2024). The present study showed that tibia bone in broiler chickens fed 20% BCAA had greater weight, strength, and calcium than that of BCAA-deprived low-protein chickens. The increase in bone strength is consistent with the enhanced deposition of the organic and inorganic matrix, likely mediated by the upregulation of osteogenic genes such as *RUNX-2, ALP*, and *BGLAP* (Table 4). The observed improvements in bone weight further indicate increased bone mass and mineral content, reflecting more robust skeletal development (Rodriguez-Navarro et al., 2018). These findings align with previous studies demonstrating that essential amino acids, particularly leucine, can stimulate osteoblast activity through the mTOR signaling pathway, leading to improved bone formation and structural integrity (Martin et al., 2020; Lv et al., 2022). Farran and Thomas (1992) investigated the impact of valine deficiency and BCAA-deprived diets on male broilers over a 3-week period, finding reduced bone ash and calcium levels in birds fed a valine-deficient diet (0.63%) compared to both control and BCAA-deficient groups. The researchers suggested that an appropriate balance of BCAA is essential for osteoblastic function, and that disrupting this balance, particularly by lowering valine, could elevate osteoclastic activity and cause bone abnormalities (Xie et al., 2025). These results suggest that BCAA supplementation supports skeletal development by enhancing both the structural (weight and strength) and compositional (mineral) properties of bone, providing a nutritional strategy to improve bone quality in rapidly growing broiler chickens.

Our results demonstrated that supplementation of BCAAs at 20% above the requirement in low-protein diets significantly improved intestinal morphology. Birds receiving the LCP+20BCAA diet exhibited the greatest villus height, villus surface area, and VH/CD ratio, while also showing an increase in goblet cell numbers and muscularis layer thickness compared with other treatments. These morphological improvements suggest a more efficient absorptive surface and enhanced mucosal protection. Consistent with our findings, Khalilzadeh et al. (2025) and Oliveira et al. (2024) also reported that dietary supplementation with BCAAs increased villus length and villus-to-crypt ratio in broilers, while enhancing mucin secretion and epithelial integrity. Similarly, Chang et al. (2015) showed that leucine supplementation improved villus height and nutrient absorption capacity in low-protein diets, supporting the beneficial role of BCAAs in gut development. BCAAs (leucine, isoleucine, valine) play pivotal roles beyond their classical function as substrates for protein synthesis, exerting regulatory effects on intestinal development and epithelial integrity (Zhou et al., 2018). One key mechanism is the activation of the mTOR signaling pathway, particularly by leucine, which promotes enterocyte proliferation, villus elongation, and enhanced protein translation in intestinal epithelial cells (Nakajo et al., 2004). This stimulation results in higher villus height and surface area, providing a greater absorptive surface for nutrient uptake. Additionally, BCAAs act as energy substrates for enterocytes and can modulate *IGF-1* signaling, further enhancing mucosal growth (Habibi et al., 2022). The increased goblet cell numbers observed may also be linked to BCAA-induced upregulation of mucin gene expression (e.g., *MUC-2*; Table 5), which contributes to the stabilization of the mucosal barrier and protection of the villi against luminal pathogens and toxins. Together, these mechanisms explain the improvements in villus morphology and VH/CD ratio observed in birds receiving supplemental BCAAs. Another important pathway involves the modulation of intestinal immune responses and oxidative status. BCAAs serve as precursors for glutamine and alanine, amino acids essential for intestinal immune cell proliferation and antioxidant defense (Liu et al., 2023). By reducing oxidative stress and inflammatory damage within the mucosa, BCAAs indirectly support crypt-villus homeostasis, preventing excessive crypt hyperplasia while maintaining efficient cell turnover (Zhang et al., 2013). This balance results in shallower crypts relative to villus length, as indicated by the improved VH/CD ratio, reflecting a more mature and functionally efficient intestinal epithelium. Moreover, the upregulation of nutrient transporter genes (as shown in Table 6) suggests that BCAA supplementation not only enhanced structural development but also improved the functional capacity of the jejunum to absorb amino acids and other nutrients.

The present findings demonstrated that supplementation with BCAA improved bone quality, as evidenced by the increased bone strength, bone weight, and mineral concentrations, particularly calcium. These phenotypic changes were in line with the alterations observed in the expression of key genes involved in bone metabolism. Among the examined genes, *RUNX-2*, the master transcription factor for osteoblast differentiation, showed enhanced expression following BCAA supplementation. This indicates that BCAA, especially leucine, may activate the mTOR pathway and promote osteoblast proliferation and differentiation, thereby initiating bone formation (Martin et al., 2020). The elevated bone weight and density observed in this study are consistent with this molecular response. Similarly, *ALP*, a crucial marker of early bone mineralization, was upregulated. *ALP* hydrolyzes phosphate esters, providing the inorganic phosphate necessary for hydroxyapatite deposition (Vimalraj, 2020). Hence, the increased calcium and phosphorus content detected in the bone samples can be directly attributed to the upregulation of *ALP. BGLAP* (osteocalcin), a non-collagenous matrix protein synthesized by mature osteoblasts, also exhibited higher expression. This finding reflects enhanced bone maturation and mineralization, since osteocalcin is widely recognized as a marker of bone quality (Bacchetta et al., 2009). The higher levels of osteocalcin expression are in agreement with the improved mechanical strength detected in our study. Furthermore, the increased expression of *COL-1*, the gene encoding type I collagen, indicates that BCAA promoted the synthesis of the organic bone matrix (Cichocka et al., 2025). A stronger collagenous framework provides the necessary scaffold for calcium and phosphate deposition, ultimately resulting in greater bone density and strength (Gredes et al., 2012). At the upstream regulatory level, *BMP-2*, a member of the *TGF-β* superfamily and a pivotal osteogenic signaling factor, was also upregulated. *BMP-2* induction suggests that BCAA may enhance not only downstream osteoblast activity but also upstream signaling cascades responsible for skeletal development (Wu et al., 2016). Finally, *FGF-23*, a hormone secreted by osteocytes that regulates systemic phosphate and vitamin D homeostasis (Wöhrle et al., 2011), was modulated by BCAA. This suggests that BCAA may improve bone mineralization indirectly by influencing calcium–phosphate balance (Go et al., 2022). The elevated bone calcium content observed in our study supports this interpretation. These findings indicate that BCAA supplementation strengthens the osteogenic pathway (*RUNX-2, ALP, BGLAP, COL-1, BMP-2*), modulates bone resorption signaling (*RANKL*), and adjusts systemic phosphate regulation (*FGF-23*). The consistency between gene expression profiles and phenotypic outcomes (enhanced bone strength, weight, and mineralization) demonstrates that the effects of BCAA on bone are not limited to nutritional aspects but extend to molecular regulation of bone metabolism. Furthermore, these results may be linked to the intestinal morphology component of this study. Improved nutrient absorption capacity in the intestine, supported by alterations in villus structure and the expression of barrier- and nutrient transport-related genes, could increase calcium and phosphorus availability for bone deposition. Thus, the effects of BCAA on bone and intestine appear to be complementary, ultimately enhancing skeletal development in broiler chickens.

The results of the present study indicated that supplementation with BCAA improved intestinal morphology, as reflected in greater villus height and villus:crypt ratio. These phenotypic outcomes were partly supported by the expression of genes associated with intestinal barrier function. In particular, the upregulation of *OCLN, ZO-1*, and *CLDN-1* suggests that BCAA strengthened tight junction integrity, thereby reducing paracellular permeability and enhancing nutrient absorption (Abtahi et al., 2023). Previous studies have similarly reported that nutritional interventions or functional feed additives improved the expression of *OCLN* and *ZO-1*, leading to tighter epithelial barriers and better growth performance in broilers (Alhotan et al., 2021; Kpodo et al., 2024; Csernus et al., 2025). Moreover, the increased expression of *MUC-2*, a marker of goblet cell activity and mucus layer thickness (Tawiah et al., 2018), implies that BCAA supplementation may have enhanced the protective mucosal layer, which not only shields epithelial cells from luminal insults but also creates a favorable environment for efficient nutrient utilization (Mendes-Frias et al., 2025). In particular, *CLDN-2*, often considered a pore-forming claudin that increases intestinal permeability (Ganapathy et al., 2022), showed less consistent regulation, and *ZO-2* expression was not markedly affected. Nevertheless, the lack of statistical significance in these genes does not undermine the overall improvement in intestinal morphology, since the upregulation of key tight junction proteins and mucin production provides sufficient evidence of enhanced barrier function. Our findings suggest that BCAA supplementation exerts positive effects on intestinal development by reinforcing epithelial integrity and mucosal defense, thereby facilitating optimal nutrient absorption to support bone mineralization and growth performance.

The present study demonstrated that dietary supplementation with BCAA significantly upregulated the expression of three critical intestinal amino acid transporters: *SLC3A1, SLC6A14*, and *SLC7A6*. These transporters are directly responsible for the absorption of leucine, isoleucine, and valine in the small intestine (Fotiadis et al., 2013). The enhanced expression of *SLC3A1* likely improved the uptake of BCAA through its heavy-chain complex (Palacín and Kanai, 2004), while *SLC6A14*, as a broad-spectrum Na+/Cl− dependent transporter, facilitated the transport of neutral and cationic amino acids, further supporting cellular uptake (Sikder et al., 2020). *SLC7A6*, involved in the coordinated transport of cationic and neutral amino acids, complements these processes to ensure efficient intestinal absorption (Verrey et al., 2004). The coordinated increase in *SLC3A1, SLC6A14*, and *SLC7A6* likely strengthened the link between nutrient supply and metabolic demands in rapidly growing broilers. By efficiently transporting BCAA into enterocytes, these transporters not only support protein synthesis and cellular energy metabolism but also may indirectly contribute to improved mineral absorption and skeletal development, as the availability of amino acids is critical for both tissue growth and bone formation (Xie et al., 2025). These findings highlight the specificity and sensitivity of key BCAA transporters to dietary supplementation and underscore their central role in mediating the physiological benefits of BCAA in broiler chickens.

## Conclusion

Dietary supplementation of BCAAs in low-protein diets enhanced intestinal morphology and barrier function through the upregulation of tight junction proteins and mucin genes. Simultaneously, the activation of bone metabolism–related genes indicated that improvements in tibial strength and quality were indirectly driven by enhanced nutrient availability and transport, as supported by the upregulation of amino acid transporter genes. Collectively, these molecular and morphological adaptations converged to improve growth performance and skeletal integrity in broilers, demonstrating the compensatory potential of BCAAs under protein-restricted conditions.

## Funding

This research was undertaken with the personal funding of the authors.

## Disclosures

The authors declare that they have no known competing financial interests or personal relationships that could have appeared to influence the work reported in this paper.

## Data Availability

Data is available upon request.
